# A qPCR assay for the rapid and specific detection of Shining ram’s-horn snail (*Segmentina nitida*) eDNA from Stodmarsh National Nature Reserve, UK

**DOI:** 10.1371/journal.pone.0288267

**Published:** 2023-11-15

**Authors:** Helen C. Rees, Mags E. Cousins, Claire A. Baker, Ben C. Maddison

**Affiliations:** 1 ADAS Biotechnology, Beeston, Nottingham, United Kingdom; 2 Natural England, Peterborough, United Kingdom; Niigata University of Pharmacy and Medical and Life Sciences, JAPAN

## Abstract

*Segmentina nitida* Müller 1774 is a freshwater snail which was formerly widespread throughout England and south Wales. Since the 1840s it has seen a rapid decline in its range which has been attributed to deteriorating water quality due to nutrient enrichment, lowering of water tables and over-management of the ditches in which it resides. *S*. *nitida* has therefore been identified as a UK Biodiversity Action Plan (UKBAP) priority species which recommends further research for its conservation. Here we have developed a Taqman based qPCR eDNA assay for the detection of *S*. *nitida* at the Stodmarsh National Nature Reserve and compared the results with a manual survey of the ditches at this location. 32 ditches were surveyed in November 2020 (22 at Stodmarsh) and February 2021 (10 outside the known range of *S*.*nitida*). Our eDNA analysis exhibited an observed percentage agreement of 84% with a kappa coefficient of agreement between manual and eDNA surveys of 0.56 (95% CI 0.22 to 0.92). Three ditches determined to be negative for *S*. *nitida* by eDNA analysis were manual survey positive, and a further two ditches that were negative by manual survey were positive by eDNA analysis revealing the potential for improved overall detection rates using a combination of manual and eDNA methodologies. eDNA analysis could therefore augment manual survey techniques for *S*. *nitida* as a relatively quick and inexpensive tool for collecting presence and distribution data that could be used to inform manual surveys and management of ditches.

## Introduction

Management and conservation of rare or at-risk species requires knowledge of species distribution via the detection of populations which could be at low densities. Traditionally, population monitoring is carried out by visual detection, identification, and counting. However, for the last decade environmental DNA (eDNA) has been used as a non-invasive sampling technique as it is reliable, cost effective, less harmful to the ecosystem and correlates well with conventional survey results [1,2].

eDNA describes the DNA that is present within an environmental sample for example water, soil, sediment, or air. The eDNA present in an environment includes DNA that originates from sloughed cellular material (e.g. skin cells) or that is excreted (e.g. faeces and urine) or secreted (e.g. saliva) by organisms occupying the environment in question [3]. Similarly, the DNA of organisms that visit the environment can also be present, for example birds or mammals drinking from a water body. The presence of an organism’s DNA within a water body is short lived as DNA has been shown to be degraded to undetectable levels within days to weeks and is affected by UV light, pH and microbial activity [4–6]. This suggests that the detection of an organism’s DNA is a demonstration of its presence or very recent presence and is a suitable surrogate for the detection/capture of the organism itself [7,8]. The analysis of eDNA by sensitive PCR-based approaches, therefore, has become an important tool for measuring species presence/absence and has been successfully used to detect many aquatic species including the highly invasive *Potamopyrgus antipodarum* New Zealand mud snail within rivers [9,10].

**Fig 1:**
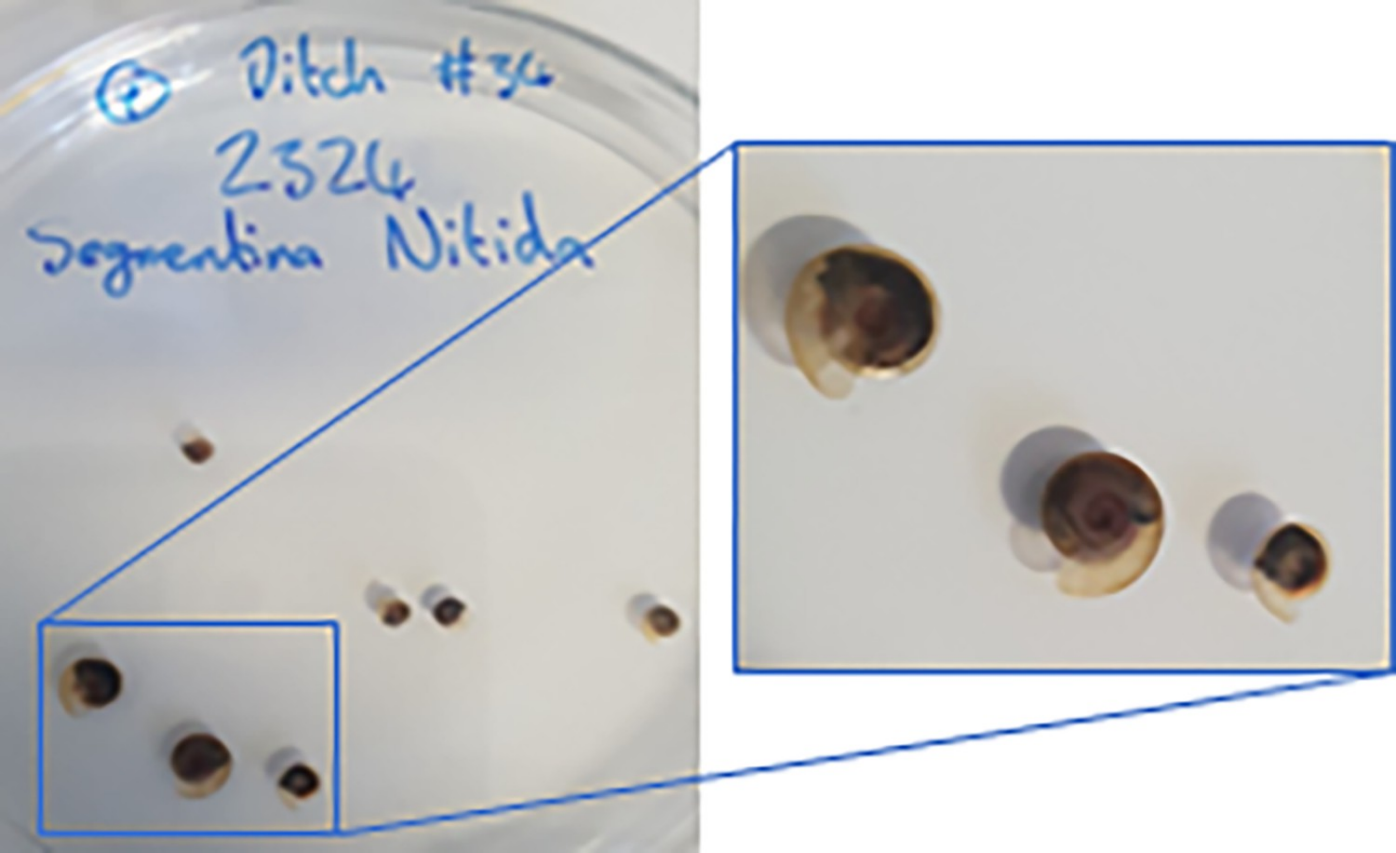
*Segmentina nitida* within a 10cm petri dish © Claire Baker.

The Shining ram’s-horn snail *Segmentina nitida* (Muller, 1774) is a small (4–6 mm diameter) rare European freshwater snail (Fig 1) which is highly sensitive to changes in management of ditches and is found only in good quality, well vegetated ditches [11]. Although once common in the UK, and widespread across lowland England and south Wales, the range of this species has substantially declined since the 1840s [12]. The decline of this species has been attributed to over-dredging of drainage ditches for land management; the conversion of grazing marshes to arable farmland; and eutrophication [13].

Despite the relative rarity of *S*. *nitida*, little is known of its ecological requirements and ecology beyond that it typically occurs in the drainage ditches of lowland grazing marshes [14]. Its presence has been verified at only a few locations in southeast England where it is mainly confined to marshes and shallow calcareous drainage ditches with dense emergent vegetation and little open water typifying ditches that are at the advanced stages of vegetation succession [14]. Watson and Ormerod further found that ditches occupied by *S*. *nitida* had significantly higher alkalinity, calcium, chloride and conductivity than unoccupied ditches and that *S*. *nitida* was absent from ditches containing elevated nitrate and nitrite levels (effects of eutrophication) despite the ditches being otherwise suitable.

The Stodmarsh National Nature Reserve (NNR) is a unique area of wetland made up of marshes, reedbeds, lakes and woodland and is home to a wide variety of wildlife especially water birds. *S*. *nitida* is listed as a rare and declining Section 41 species (species which are of principal importance for the conservation of biodiversity in England and are listed under Section 41 (S41) of the 2006 Natural Environment and Rural Communities (NERC) Act) and also a Ramsar criteria feature of the Stodmarsh NNR and to provide sensitive management of ditches at this site, there is a requirement to know where the *S*. *nitida* occurs. In 2019, the Natural England Field Unit carried out a trial manual survey of 23 ditches and found the presence of *S*. *nitida* in seven of these ditches which included five ditches where it had not been previously known to occur [15]. Although an encouraging result this highlighted the importance of knowing the whereabouts of existing colonies to allow a ditch management programme to be developed that accounted for these occurrences.

This study therefore set out to develop an eDNA based assay for the detection of *S*. *nitida* which could be used to inform and complement traditional manual surveys at the Stodmarsh NNR. Here, we describe a qPCR assay for the detection of *S*. *nitida* and demonstrate the detection of this species within ditch water samples from Stodmarsh NNR with known presence/absence status.

## Materials and methods

### Sampling design

**Fig 2:**
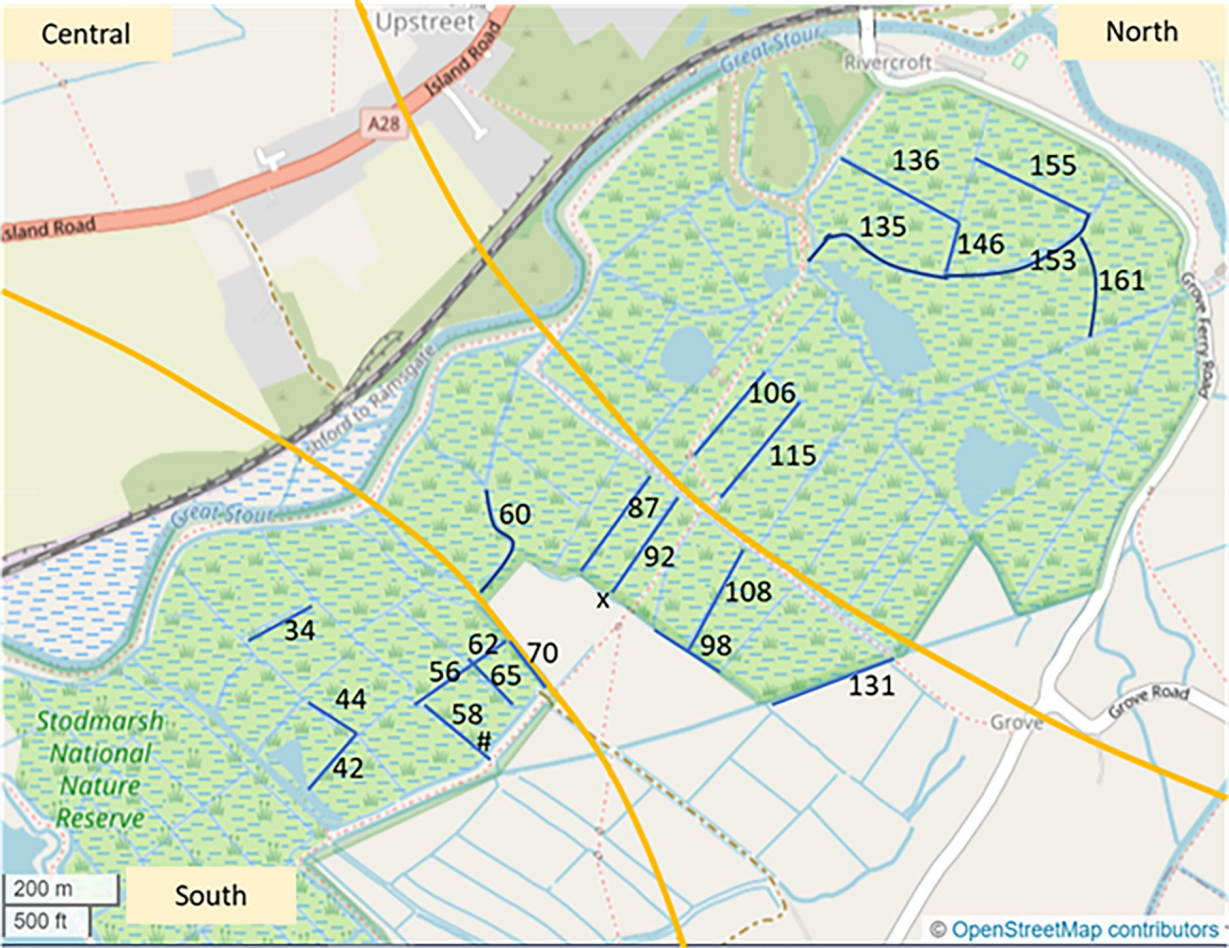
Map of ditch locations within the Stodmarsh NNR, Kent. Yellow lines denote the three hydrological units: North, Central, and South. Sampled ditches are marked in blue. Priority ditches (presence found in 2019 survey; Cousins, 2020) were 87, 92, 135, 136, 146 and two unnumbered ditches one known as Metal bridge ditch (approximate location marked with an ‘x’) and the other located at the south-eastern end of ditch 65 (approximate location marked with a ‘#’). OpenStreetMap^©^ (data available under the Open Database License).

The hydrology of the Stodmarsh NNR is complex, however, *S*. *nitida* is known to occupy all three major hydrological units (Fig 2) so any differences between the hydrology of the three units does not prohibit the snail occupying at least some of the ditches in each of the major units. At certain times there are connections between all three hydrological units, and there are some control points on the site where the connections can be managed. During the survey effort, water levels were rising therefore it was important to understand the possible linkages between the ditches and how these could impact findings.

The ditches targeted for eDNA sampling were eight ditches where there were previous records for *S*. *nitida* (Fig 2), plus connected and nearby ditches, up to a total of 22. eDNA sampling effort aimed to obtain samples from all three major hydrological units (Fig 2, labelled north, central and south) where the snail was known to occur, to obtain adequate representation of occupied areas of the site. eDNA surveyors were instructed to sample up to a total of 7–8 ditches in each of the north, central and south hydrological units, and more than 8 ditches were highlighted on their plans in each hydrological unit to give surveyors some choice in case some were too dry or inaccessible.

An aquatic invertebrate survey of the ditches, targeting those which had already been sampled for eDNA was carried out subsequent to eDNA sample collection. This was commissioned to determine presence/absence of *S*. *nitida* in those ditches to enable comparison of traditional/manual sampling with the results of the eDNA method.

### Sample collection

22 ditch water samples were collected from ditch surface water by Natural England staff at Stodmarsh NNR between the 16^th^ and 27^th^ November 2020 (Fig 2). 10 ditch water samples outside the known range of S. nitida were collected by ADAS staff between the 10^th^ January and the 4^th^ February 2021 (sample information can be found in S1 and S2 Tables and S1 Protocol). Each sample was collected using its own dedicated sampling pack i.e. sterile disposable gloves, a sterile scoop, a sterile sampling bag, and a sterile 50ml syringe. 20x 30 mL water samples were collected along the length of each of the ditch and pooled into sterile sampling bag. Up to 500 mLs of this water was filtered through a 0.22 μm PES sterivex filter (Millipore) using a 50 mL luer-lock syringe (S1 Table). After filtration of water, 95% ethanol was added as a preservative. One control field blank (distilled water) was also filtered by Natural England staff on site.

Snail specimens were collected during an invertebrate survey carried out at the same 22 ditches at Stodmarsh NNR by The Ecology Co-op, contracted by Natural England between December and January 2020/2021 (specimen information and detailed sampling methodology can be found in S3 Table). Manual surveys of the 22 ditches were performed using an adapted invertebrate sampling protocol from [16]. The emphasis was on the free-style netting of suitable looking micro-habitats (e.g. emergent vegetation stands) that are likely to be most productive for this assemblage. Effort was deliberately not divided in proportion to the extent of features nor length of ditch, since species are not distributed in this fashion. Snail specimens from *S*. *nitida* and nine other similar ram’s-horn shaped species, from the 16 species of snail found during invertebrate survey were identified and preserved in 95% ethanol prior to shipment to the laboratory: *Anisus vortex*, *Bathyomphalus contortus*, *Gyraulus crista*, *Gyraulus albus*, *Hippeutis complanatus*, *Planorbarius corneus*, *Planorbis carinatus*, *Planorbis planorbis*, *Segmentina nitida*, *Valvata cristata*.

### DNA extraction

Upon return to the laboratory, the preservative solution was removed from the sterivex filters and any DNA containing material captured on the filter membrane was recovered by addition of 720 μL of ATL lysis buffer and 40 μl proteinase K to the filter, followed by incubation at 56°C in a water bath with regular mixing by vortexing. The supernatant was then extracted using the DNeasy blood and tissue kit (Qiagen) following the manufacturer’s instructions with final resuspension in 200μl of elution buffer. All DNA samples were quantified using a Qubit 3.0 Fluorometer (Invitrogen) following the manufacturer’s instructions then stored at -20 ⁰C before use. Field blanks (x1) and extraction blanks (x2) were also processed alongside the Stodmarsh sterivex filters. All eDNA extractions from filters were performed in a separate laboratory remote from snail specimen eDNA extraction and qPCR set up, using dedicated tissue extraction equipment. Disposable laboratory coats were worn, and benches and equipment were wiped down with a 10% bleach solution before and after use.

Each snail specimen was individually transferred to a clean, sterile mortar and ground into a fine paste using a pestle and liquid nitrogen. For some snail species, the individual specimens were pooled prior to grinding. After use mortar and pestles were immediately immersed in 10% bleach for a minimum of 10 minutes and then cleaned in between samples with 10% Distel (Tristel™), rinsed with dH_2_O and then autoclaved at 121 ⁰C for 15–20 minutes. DNA was extracted by the DNeasy blood and tissue kit (Qiagen) -with a final elution volume of 50 or 200μl—in a separate laboratory remote from water sample extraction and qPCR set up, using dedicated tissue extraction equipment. Disposable laboratory coats were worn, and benches and equipment were wiped down with a 10% bleach solution before and after use. Extracted DNA was quantified using a Qubit 3.0 Fluorometer (Invitrogen) using the Qubit 1X dsDNA BR Assay Kit following the manufacturer’s instructions then stored at -20°C before use.

### Snail specimen identification

All PCR set up was performed in a clean ‘PCR room’ within a UV sterilisable cabinet and in a separate laboratory to DNA extraction using dedicated equipment and PPE. To ensure the unidirectional workflow DNA extracts are collected from the DNA extraction laboratory and transferred to the PCR set-up laboratory. Laboratory personnel do not return to the DNA extraction laboratory during that same day thus maintaining the unidirectional workflow.

PCR was performed to confirm the identity of the provided snail specimens using the mICOIintF/jgHCO2198 primer combination [17] (S1 Fig for primer location on *S*. *nitida* sequences). These primers amplify a fragment of the Cytochrome c Oxidase subunit I gene (COI) and have been shown to perform well in invertebrate metabarcoding studies [17,18]. PCRs were set up in a total volume of 25 μL consisting of: 2 μL of extracted template DNA, 2.5 μL of each primer (final concentration 0.4 μmol/L), 12.5 μL of Itaq (BioRad) Sybr Green mastermix, and 5.5 μL ddH2O. Each sample was run in duplicate on a Bio-Rad CFX Connect real-time PCR machine as follows: an initial incubation for 1 minute at 95⁰C; followed by 35 cycles with a melting temperature of 95°C for 1 minute; an annealing temperature of 40⁰C; an extension step at 72°C for 30 seconds; followed by a final extension step at 72⁰C for 90 seconds before holding at 4⁰C until collection of PCR products for analysis. After PCR and amplicon clean-up using the Macherey-Nagel™ NucleoSpin™ Gel and PCR Clean-up Kit according to the manufacturers instructions, PCR products were Sanger sequenced using mICOIintF and returned sequences identified using BLAST.

### Species-specific assay development

In order to design primers specific to *S*. *nitida* the DNA sequences for the cytochrome oxidase 1 (COI) gene for *S*. *nitida* and the nine other co-occurring similar ram’s-horn shaped species commonly found at the Stodmarsh NNR were downloaded from Genbank and their sequences aligned using BioEdit version 7.2.5. Primers and probes were designed using PrimerBLAST with default settings except for targeting a 70–300 bp fragment and including only base pairs between 70 and 600 in the *S*. *nitida* consensus sequence generated from the barcode region of the COI as this corresponded to the most variable region on the multi-species alignment. Ten potential primer/probe combinations were generated and reduced to four using PrimerBLAST and looking for cross-species amplification (S5 Table).

The four potential primer/probe combinations were tested firstly on DNA extracted from *S*. *nitida* followed by the other nine co-occurring snail species to test for cross-species reactivity. PCRs were set up in a total volume of 25 μL consisting of: 1 μL of each primer/probe (final concentrations: 0.2 μmol/L forward primer; 0.4 μmol/L reverse primer; 0.1 μmol/L probe), 12.5 μL of TaqMan® Environmental Master Mix 2.0 and 6.5 μL ddH2O. 3μL of DNA was added to each reaction and 12 replicates were performed per DNA sample. All controls were performed in quadruplicate with a dilution series of 1x10^-1^ to 1x10^-4^ ng/μL *S*. *nitida* DNA being used as positive controls and ddH2O in place of DNA for the negative control. PCRs were run on a Bio-Rad CFX Connect real-time PCR machine as follows: an initial incubation for 5 minutes at 56.3⁰C then 10 minutes at 95°C; followed by 55 cycles with a melting temperature of 95°C for 30 seconds and an annealing temperature of 59.6⁰C for 1 minute.

Once specificity of primer/probe combinations was confirmed, the primer concentrations of two primer/probe combinations (primer/probe combinations 2 and 9) were optimised by independently varying final primer concentrations (the probe was held at a final concentration of 0.1 μmol/L) [19]. The sensitivity of the assay was tested by creating a six-level standard curve dilution series (3x10^-1^ to 3x10^-7^ μg/μl). The standard curve was created by quantifying the DNA extracted from *S*. *nitida* sample 7a on a Qubit Fluorometer (Thermo Fisher Scientific) and diluting the DNA to the desired concentrations using the elution buffer provided in the DNeasy Blood and Tissue kit (Qiagen). 12 replicates of each dilution were run using the optimised primer/probe concentrations to determine the standard curve slope, the limit of detection (LOD) and limit of quantification (LOQ).

The optimised assay using primer/probe combination 9 (Snit9F: 5’- CCACTTTTAATTGGGGCTCCG-3’; Snit9R: 5’- CCATGTGCAATAGGACCGCT-3’; Snit9P: 5’- TGAAGGAGGTGTTGGTACTGGGTG-3’, FAM/BHQ-1) (S2 Fig) was used to determine the presence/absence of *S*. *nitida* within the 22 ditch samples from Stodmarsh NNR and the 10 ditch samples from outside of the known range of *S*. *nitida*. A subset of amplified products were sent for Sanger sequencing to confirm that the target species have been amplified.

### Inhibition testing

All DNA extracts were tested for inhibition by adding 3μL of extract into a qPCR for the amplification of an internal positive control (DNA derived from the plasmid pSD3). PCRs were set up in a total volume of 25 μL consisting of: 3 μL of internal positive control DNA (0.08 ng/μl),1 μL of each primer/probe (final concentraions: 0.2 μmol/L forward primer; 0.4 μmol/L reverse primer; 0.1 μmol/L probe; Supplemental file 2), 12.5 μL of TaqMan® Environmental Master Mix 2.0 (containing AmpliTaq GOLD DNA polymerase), and 3.5 μL ddH2O. 3μL of extracted DNA template was added to each reaction and 12 replicates were performed per DNA sample. All controls were performed in quadruplicate with a dilution series of 0.08 ng/μl to 0.0032 ng/μL internal positive control DNA being used as positive controls and ddH2O in place of DNA for the negative control. PCR cycling was as above.

### Inhibitor removal

Two of the samples were found to be completely inhibited therefore these were put through an inhibitor removal process using the OneStep PCR inhibitor removal kit (Zymo Research) according to the manufacturers’ instructions. The inhibition and *S*.*nitida* testing was then repeated for these two samples.

### Statistical analysis

To measure the agreement between the two survey methods, that is, manual survey and eDNA analysis, Cohen’s kappa coefficient [20] was calculated as follows:

$$k=\frac{\mathrm{Pr}\left(a\right)-Pr(e)}{1-Pr(e)}$$

where Pr(a) is the relative agreement among rates, and Pr(e) is the hypothetical probability of chance agreement, using the observed data to calculate the probabilities of each method randomly giving a positive detection. If the methods are in complete agreement, then *k* = 1. If there is no agreement other than what would be expected by chance, *k* = 0.

## Results

### Detection

The results of the manual survey are shown within the S4 Table. Collected snail specimens were confirmed as the taxonomically identified species (S4 Table) by Sanger sequencing of PCR products with identities of at least 98% over the length of the sequence. The exception to this was the potential *Valvata cristata* specimens where the sequences only showed 96 or 97% identity to those on the NCBI database with the sequences also being found to be similarly close to those of *Valvata relicta* which is a species not found in the UK and is therefore unlikely to be present at Stodmarsh. Six additional species were potentially found during manual survey, but specimens were not collected largely as the surveyor was requested to collect specimens of small freshwater snails and these constituted larger species e.g. *Bithyia tentaculata* (8-12mm high, 6-7mm wide).

Only DNA from *S*. *nitida* specimens was found to amplify using the selected primer combination (primer/probe combination 9) i.e. there was no cross-species reactivity with the other snail species present at Stodmarsh and the assay was specific for *S*. *nitida*. The remaining six species found during manual survey were found not to amplify during *in silico* analysis.

The limit of detection (LOD) and limit of quantification (LOQ) of primer/probe combination 9 were both found to be 3x10^-4^ ng/μL. The LOD and LOQ have various definitions in the eDNA literature, here LOD is defined as the lowest standard concentration at which 95% of technical replicates amplify and LOQ is the lowest standard concentration for which the coefficient of variation (CV; equal to the standard deviation quantity divided by the mean quantity of a group of replicates) value is <35% [21]. The LOD corresponded to a Ct value of 37.47 which encompassed 100% of the Ct values in this study i.e. all positive amplifications were above the limit of detection. Detection in a water sample was indicated by at least 1 of 12 positive qPCR replicates [1].

All Stodmarsh NNR ditch water samples and ditch samples from outside the known range of *S*. *nitida* were subjected to inhibition testing, all samples except two from Stodmarsh NNR (136 and 146) did not cause inhibition of qPCR. Samples 136 and 146 caused complete inhibition of the qPCR inhibition assay however after inhibitor removal no longer caused inhibition of qPCR inhibition assay. After inhibitor removal the two samples were retested using the *S*. *nitida* qPCR but were still found to be negative for target species.

All Stodmarsh NNR ditch water samples and ditch samples from outside the known range of *S*. *nitida* were then subjected to the optimised assay for *S*. *nitida* detection, with seven ditches from Stodmarsh NNR being positive for *S*. *nitida* DNA (Table 1). One ditch (ditch 34) with very low positivity (1/12), was re-tested to confirm positivity (3/12 on repeat). All remaining ditches were negative for *S*. *nitida* DNA, therefore other than those ditches where *S*. *nitida* was found by manual survey—ditches 62, 70 and 108 (Table 1)—*S*. *nitida* is likely to be absent.

**Table 1:** Summary of *S*. *nitida* survey and PCR status of the 32 ponds studies.

Sample ID	DNA (ng/μL)	*S*. *nitida* species-specific qPCR result	*S*. *nitida* manual survey
1	<1 ng/μL	0/12; negative	N/A
2	<1 ng/μL	0/12; negative	N/A
3	<1 ng/μL	0/12; negative	N/A
4	<1 ng/μL	0/12; negative	N/A
5	<1 ng/μL	0/12; negative	N/A
D1	<1 ng/μL	0/12; negative	N/A
D2	<1 ng/μL	0/12; negative	N/A
Top	1.7	0/12; negative	N/A
LHS	1.34	0/12; negative	N/A
RHS	<1 ng/μL	0/12; negative	N/A
34	2.14	1*/12; positive	positive
42	2.95	0/12; negative	negative
44	1.71	0/12; negative	negative
56	6.56	12/12; positive	positive
58	11.1	0/12; negative	negative
60	3.96	0/12; negative	negative
62	2.11	0/12; negative	positive
65	3.78	9/12; positive	positive
70	0.84	0/12; negative	positive
87	18.1	12/12; positive	positive
92	4.88	6/12; positive	positive
98	4.36	3/12; positive	negative
106	1.66	0/12; negative	negative
108	2.32	0/12; negative	positive
115	2.06	4/12; positive	negative
131	1.08	0/12; negative	negative
135	1.46	0/12; negative	negative
136	<1 ng/μL	0/12; negative	negative
146	<1 ng/μL	0/12; negative	negative
153	1.89	0/12; negative	negative
155	<1 ng/μL	0/12; negative	negative
161	1.35	0/12; negative	negative
Positive control	5 ng/μl	N/A	N/A
Negative control	n/a	N/A	N/A

Samples marked as ‘<1 ng/μL were samples where the DNA concentration was too low to measure using the Qubit broad range kit. Green highlighted samples were found to be positive for S. nitida by both qPCR and manual survey; orange highlighted surveys were found to be positive for S. nitida by qPCR but not manual survey; red highlighted samples were found to be positive for S. nitida by manual survey but not by qPCR.

*qPCR was repeated for this sample to confirm positivity and 3/12 replicates were found to be positive for S. nitida.

### Statistical analysis

Using the observed percentage agreement of the two methods of 0.84 (1 = 100%) and the probability of random agreement of 0.64, Cohen’s kappa coefficient was calculated as 0.56 (95% CI 0.22 to 0.92) for manual survey-positive ponds versus their qPCR analysis results i.e. moderate agreement.

## Discussion

This study was carried out to design and evaluate the use of an eDNA assay for the detection of *S*. *nitida* in ditches at Stodmarsh NNR and to compare to manual survey data taken shortly after water samples were collected. *S*. *nitida* DNA was detected at 62.5% (5 of 8) of the ditches where *S*. *nitida* presence was detected by manual survey and in two additional ditches where *S*. *nitida* was not detected by manual survey (7 ditches in total). A further 22 ditches sampled within Stodmarsh NNR and outside the known distribution of *S*. *nitida* were negative for *S*. *nitida*. This resulted in an observed percentage agreement of 84% and a kappa coefficient of 0.56 which shows a moderate agreement between the results. When taken together, manual survey in combination with eDNA analysis has led to improved *S*. *nitida* detection rates, that is, 10 of 32 ditches rather than the 8 reported by field survey. However, it still should be noted that where eDNA was detected but no *S*. *nitida* was found by manual survey (ditches 98 and 115) it is unknown whether the animals were present but not detected by manual survey (present in very low numbers), or whether they are absent and the eDNA drifted into the ditch e.g. through hydrological connections. Ditches 98 and 115 are not directly adjacent to occupied ditches and the direction of flow would not ‘normally’ facilitate drift between the ditches concerned in normal flow conditions, but ultimately they are connected nevertheless.

Where rare or threatened species are concerned, it is likely that their detection by either manual survey or eDNA will be imperfect leading to an underestimation of its distribution.

During particular time periods or developmental stages, some species can be difficult to detect potentially biasing survey outcomes [22,23]. This may have been the case here for the two ditches where *S*. *nitida* was found by manual survey but not via qPCR of eDNA. A study in Poland showed that *S*. *nitida* breed during April-May [24] and although conditions may be slightly different in the UK as these ditch water samples were taken outside the likely breeding season their detection will be more difficult as there is likely to be less *S*. *nitida* DNA in the water. The sampling strategy of taking 20x 30 mL samples along the length of each ditch should have overcome the fact that eDNA can be highly localised in space and time [25]. To achieve a higher level of coverage (especially for longer ditches), more samples may need to be taken. This could allow for targeting more locations within the ditches where *S*. *nitida* would most likely be present, that is, where the ditch is thickly vegetated, thus improving the probability of detection.

A further cause of false negative results can be PCR inhibition [26,27]. Upon testing of the DNA extracts two samples, from ditches 136 and 146, were found to cause complete inhibition of the qPCR inhibition assay (although inhibition was removed after sample clean-up). It was therefore unlikely that inhibition was the reason for the qPCR of the samples from ditches 62, 70 and 108 being negative for *S*. *nitida*.

It is also likely that the volume of water filtered will play an important role in the detection of *S*. *nitida*. The volume of water filtered was between 200 mL and 500 mL for all samples—the volume determined by how soon the filter clogged. A larger pore sized filter e.g. 0.45μM or 0.8μM or the use of a pre-filter could be used in future studies. It is common for volumes of water between 500 mL and 5L to be filtered although there is little consensus on the minimum volume [28]. Small volumes (0.25 L) have been shown to contain detectable eDNA from macroinvertebrate species when a range of volumes up to 2 L were sampled and analysed [29] and increasing the volume of filtered water has been shown to have a positive effect on eDNA capture and PCR amplification efficiency [30]. However, although a larger pore size filter allows an increased volume of water to be filtered, the smallest particles containing eDNA may pass through the filter. The alternative, pre-filtration can increase single species detection probability [31] and give more consistent results for community compositions. However, the use of pre-filters can lower the DNA yield and the number of detected taxa [32] therefore there is a trade-off to be made when deciding on the filter to be used.

Finally, the density of snails in the ditch system will play an important role in its detectability. The relative abundance of *S*. *nitida* was recorded during the manual survey (Supplemental file 1), and at sites where the eDNA assay was negative for *S*. *nitida* but it was found during manual survey, the abundance was recorded as occasional or rare. Studies on the New Zealand mud snail have shown that this snail’s eDNA can be detected when snails are present at low densities [9], this species is similar in size to *S*. *nitida*, however, far larger volumes of water (4 L) were filtered (using multiple filters if necessary) prior to analysis which could account for its detectability at low density although eDNA transport would need to be considered as these samples were taken from a river system.

Since this study was carried out a study [13] investigating the population structure of *S*. *nitida* individuals from Poland, Germany, Sweden and the UK to identify differences both within and between populations has been published. The study found that there are two distinct genetic lineages (also distinct in shape), one in western Europe (UK, Germany–Lineage 1) and one in eastern Europe (Poland, Sweden–Lineage 2). Although only a UK population was tested during the present study it likely that the assay designed herein would also detect *S*. *nitida* populations in eastern Europe as during the primer design phase the three *S*. *nitida* sequences retrieved from Genbank were from specimens collected in Poland, Denmark and Germany. Future work could involve testing our primer/probe combination on individuals from these other populations.

*S*. *nitida* was found to be present in the ditches 136 and 146 during trial surveys in 2019 [15] but not during aquatic invertebrate surveys in December/January 2020/2021 nor or via eDNA survey. It is therefore possible that the population in these ditches has been lost. To confirm the results of the eDNA assay designed herein further manual survey is required to corroborate both the loss of *S*.*nitida* from ditches 136 and 146 and the two additional eDNA assay positive ditches found. For those manual survey samples where *S*. *nitida* specimens were found but the eDNA assay did not detect *S*. *nitida* DNA a larger pore sized filter/filtration of more water may be required to enable eDNA assay corroboration. This study has shown that eDNA assays can be used for the detection of *S*. *nitida* and if used in the future could have time and cost savings and could inform manual survey and therefore management of the ditches.

## Supporting information

S1 Fig *Segmentina nitida* COI sequence alignment. Alignment shows the location of the primers used to generate A. COI barcode sequence—SnitCOIF/SnitCOIR (yellow) and B. short COI sequence for specimen identification and metabarcoding—mICOIintF/jgHCO2198 (green). Each line (LC429396.1 and EF012178.1) represents a *S*. *nitida* COI sequence from a different specimen.
(DOCX)

S2 Fig *S*. *nitida* sequence alignment showing the positions of the four potential species-specific primer/probe combinations. LC429396.1 represents an *S*. *nitida* COI sequence. Primer pair 2 and its probe shown in green; primer pair 4 and its probe shown in blue; primer pair 9 and its probe shown in red; and primer pair 10 and its probe shown in grey. Only the relevant part of the alignment is shown.
(DOCX)

S1 Table Stodmarsh NNR ditch samples. (DOCX)

S2 Table Leicestershire ditch samples. (DOCX)

S3 Table Mollusc species list as sampled by Dan Bennett (Dec 2020). The abundance attribution made by D. Bennett is qualitative. (DOCX)

S4 Table DNA information per snail species. Where there was poor PCR amplification/sequence we were unable to amplify sufficient target gene to enable us to identify the species and the specimen was not used further. *Valvata cristata* samples only showed 96 or 97% identity to those on the NCBI database with the sequences also being found to be similarly close to those of *Valvata relicta* which is not found in the UK and is therefore unlikely to be present. The sequences were manually checked versus their respective chromatograms to resolve any errors in base calling prior to identification via BLAST searches, however, no improvements were made.
(DOCX)

S5 Table Ten potential primer/probe combinations for S. nitida species-specific PCR for in silico testing. (DOCX)

S1 Protocol Collection of invertebrate samples. (DOCX)

## Abbreviations

COI: Cytochrome oxidase I
eDNA: Environmental DNA
qPCR: quantitative polymerase chain reaction
NNR: National Nature Reserve
