# Supplementary material for: A qPCR assay for the rapid and specific detection of Shining ram’s-horn snail (Segmentina nitida) eDNA from Stodmarsh National Nature Reserve, UK
Source: PLoS One. 2023 Nov 15;18(11):e0288267. doi: 10.1371/journal.pone.0288267 (PMC10651049; doi:10.1371/journal.pone.0288267)
Supplement: S1 Fig — Alignment shows the location of the primers used to generate A. COI barcode sequence—SnitCOIF/SnitCOIR (yellow) and B. short COI sequence for specimen identification and metabarcoding—mICOIintF/jgHCO2198 (green). Each line (LC429396.1 and EF012178.1) represents a S. nitida COI sequence from a different specimen. (DOCX) [file pone.0288267.s001.docx]

LC429396.1 ACCTTATATTTGATTTTTGGTGTTTGATGTGGTTTAGTTGGTACTGGTTTATCTCTATTA 60

EF012178.1 -------------------------------GTTTAGTCGGTACTGGTTTATCTCTATTA 29

LC429396.1 ATTCGTTTGGAATTAGGTACCTCTGGTGTATTAATAGATGAACATTTTTATAATGTTATT 120

EF012178.1 ATTCGTTTGGAATTAGGTACCTCTGGTGTATTAATAGATGAACATTTTTATAATGTTATT 89

LC429396.1 GTTACTGCACATGCTTTTATTATAATTTTTTTTATAGTTATACCAATAATAATTGGTGGT 180

EF012178.1 GTTACTGCACATGCTTTTATTATAATTTTTTTTATAGTTATACCAATAATAATTGGTGGT 149

LC429396.1 TTTGGTAATTGAATAATTCCACTTTTAATTGGGGCTCCGGATATATCATTTCCTCGTATA 240

EF012178.1 TTTGGTAATTGAATAATTCCACTTTTAATTGGGGCTCCGGATATATCATTTCCTCGTATA 209

LC429396.1 AATAACATATCATTCTGGTTACTACCACCATCTTTTATCCTTTTATTGATTTCTTCTATA 300

EF012178.1 AATAACATATCATTCTGGTTACTACCACCATCTTTTATCCTTTTATTGATTTCTTCTATA 269

LC429396.1 GTTGAAGGAGGTGTTGGTACTGGGTGAACTGTTTATCCCCCCTTAAGCGGTCCTATTGCA 360

EF012178.1 GTTGAAGGAGGTGTTGGTACTGGGTGAACTGTTTATCCCCCCTTAAGCGGTCCTATTGCA 329

LC429396.1 CATGGTGGTGCATCAGTTGATTTAGCTATTTTTTCATTACACTTGGCCGGTATATCTTCT 420

EF012178.1 CATGGTGGTGCATCAGTTGATTTAGCTATTTTTTCATTACACTTGGCCGGTATATCTTCT 389

LC429396.1 ATTTTAGGTGCTATTAATTTTATTACCACTGTAATAAACATGCGGGCTCCAGGTATTACT 480

EF012178.1 ATTTTAGGTGCTATTAATTTTATTACCACTGTAATAAACATGCGGGCTCCAGGTATTACT 449

LC429396.1 ATGGAACGATTATCTTTATTTGTCTGGTCTGTATTAATTACAGCATTTTTGTTACTATTA 540

EF012178.1 ATGGAACGATTATCTTTATTTGTCTGGTCTGTATTAATTACAGCATTTTTGTTACTATTA 509

LC429396.1 TCATTACCAGTTTTAGCTGGTGCCATTACAATATTATTAACGGATCGTAATTTTAATACT 600

EF012178.1 TCATTACCAGTTTTAGCTGGTGCCATTACAATATTATTAACGGATCGTAATTTTAATACT 569

LC429396.1 AGTTTCTTTGATCCAGCAGGTGGTGGTGATCCTATCTTATA------------------- 641

EF012178.1 AGTTTCTTTGATCCAGCAGGTGGTGGTGATCCTATCTTATATCAACATTTATTTTGATTT 629

LC429396.1 ------------------------------------------------------------ 641

EF012178.1 TTTGGTCATCCAGAAGTATATATTTTAATTTTACCAGGGTTTGGTATGGTTTCACATATT 689

LC429396.1 ------------------------------------------------------------ 641

EF012178.1 TTAAGTAATTTTGTTTCAAAACCAGCTTTTGGTACTTTAGGAATAATTTATGCTATAGTT 749

Figure S6. S. nitida COI sequence alignment

Alignment shows the location of the primers used to generate A. COI barcode sequence - SnitCOIF/SnitCOIR (yellow) and B. short COI sequence for specimen identification and metabarcoding - mICOIintF/jgHCO2198 (green). Each line (LC429396.1 and EF012178.1) represents a S. nitida COI sequence from a different specimen.
