# Supplementary material for: A qPCR assay for the rapid and specific detection of Shining ram’s-horn snail (Segmentina nitida) eDNA from Stodmarsh National Nature Reserve, UK
Source: PLoS One. 2023 Nov 15;18(11):e0288267. doi: 10.1371/journal.pone.0288267 (PMC10651049; doi:10.1371/journal.pone.0288267)
Supplement: S2 Fig — LC429396.1 represents an S. nitida COI sequence. Primer pair 2 and its probe shown in green; primer pair 4 and its probe shown in blue; primer pair 9 and its probe shown in red; and primer pair 10 and its probe shown in grey. Only the relevant part of the alignment is shown. (DOCX) [file pone.0288267.s002.docx]

LC429396.1 TTTGGTAATTGAATAATTCCACTTTTAATTGGGGCTCCGGATATATCATTTCCTCGTATA 240

CCACTTTTAATTGGGGCTCCG

LC429396.1 AATAACATATCATTCTGGTTACTACCACCATCTTTTATCCTTTTATTGATTTCTTCTATA 300

LC429396.1 GTTGAAGGAGGTGTTGGTACTGGGTGAACTGTTTATCCCCCCTTAAGCGGTCCTATTGCA 360

GAGGTGTTGGTACTGGGTGA GCGGTCCTATTGCA

AGGAGGTGTTGGTACTGGGTG GCGGTCCTATTGCA

TGAAGGAGGTGTTGGTACTGGGTG AGCGGTCCTATTGCA

LC429396.1 CATGGTGGTGCATCAGTTGATTTAGCTATTTTTTCATTACACTTGGCCGGTATATCTTCT 420

CATGGTGGT

CATGGTGGT

CATGG

LC429396.1 ATTTTAGGTGCTATTAATTTTATTACCACTGTAATAAACATGCGGGCTCCAGGTATTACT 480

TAAACATGCGGGCTCCAGG

ATAAACATGCGGGCTCCAGG

ATAAACATGCGGGCTCCAGG

LC429396.1 ATGGAACGATTATCTTTATTTGTCTGGTCTGTATTAATTACAGCATTTTTGTTACTATTA 540

LC429396.1 TCATTACCAGTTTTAGCTGGTGCCATTACAATATTATTAACGGATCGTAATTTTAATACT 600

ACCAGTTTTAGCTGGTGCCATTACAA

LC429396.1 AGTTTCTTTGATCCAGCAGGTGGTGGTGATCCTATCTTATA------------------- 641

AGCAGGTGGTGGTGATCCTA

Figure S8. S. nitida sequence alignment showing the positions of the four potential species-specific primer/probe combinations.

LC429396.1 represents an S. nitida COI sequence.

Primer pair 2 and its probe shown in green; primer pair 4 and its probe shown in blue; primer pair 9 and its probe shown in red; and primer pair 10 and its probe shown in grey. Only the relevant part of the alignment is shown.
