# Supplementary material for: A qPCR assay for the rapid and specific detection of Shining ram’s-horn snail (Segmentina nitida) eDNA from Stodmarsh National Nature Reserve, UK
Source: PLoS One. 2023 Nov 15;18(11):e0288267. doi: 10.1371/journal.pone.0288267 (PMC10651049; doi:10.1371/journal.pone.0288267)
Supplement: S1 Table — (DOCX) [file pone.0288267.s003.docx]

| **Sample ID** | **Collection date/time** | **Sampler** | **GPS Location** | **Volume filtered** | **Sample Condition** | **Site conditions** |
| --- | --- | --- | --- | --- | --- | --- |
| 34 | 23/11/2020; 13.00 | KO/PW | 51.315018-1.192531 | 250ml | Low turbidity | Sunny, 8 degrees |
| 42 | 23/11/2020; 14.00 | KO/PW | 51.313210-1.193633 | 450ml | Low turbidity | Sunny, 8 degrees |
| 44 | 23/11/2020; 13.30 | KO/PW | 51.313603-1.193356 | 250ml | Low turbidity | Sunny, 8 degrees |
| 56 | 23/11/2020; 11.30 | KO/PW | 51.313864-1.196120 | 400ml | Low turbidity | Sunny, 7 degrees |
| 58 | 23/11/2020; 10.55 | KO/PW | 51.313686-1.196216 | 300ml | Low turbidity | Sunny, 7 degrees |
| 60 | 17/11/2020; 14.00 | KO/PW | 51.315713-1.197350 | 62/1: 200ml; 62/2: 150ml | Low turbidity | Sunny spells, 14 degrees |
| 62 | 17/11/2020; 14.30 | KO/PW | 51.314608-1.197528 | 62/1: 200ml; 62/2: 150ml | Low turbidity | Sunny spells, 14 degrees |
| 65 | 23/11/2020; 14.50 | KO/PW | 51.313703-1.198258 | 65/1: 70ml, 65/2: 70ml, 65/3: 70ml | High turbidity | Cloud cover, 7 degrees |
| 70 | 17/11/2020; 13.10 | KO/PW | 51.314726-1.197878 | 500ml | Low turbidity | Sunny, 14 degrees |
| 87 | 18/11/2020; 11.55 | KC/KO | 51.316071-1.200108 | 350ml | Low turbidity | Sunny, windy |
| 92 | 18/11/2020; 10.58 | KC/KO | 51.316634-1.201841 | 92/1: 170ml; 92/2: 150ml | Low turbidity | Sunny, breezy, warm |
| 98 | 18/11/2020; 14.00 | KC/KO | 51.314733-1.201146 | 98/1: 100ml; 98/2: 100ml | Medium turbidity | Windy, overcast |
| 106 | 27/11/2020; 13.00 | KO/PW | 51.317576-1.202240 | 400ml | Low turbidity | Overcast, 6 degrees |
| 108 | 18/11/2020; 13.00 | KC/KO | 51.314511-1.202374 | 108/1: 100ml; 108/2: 100ml | Medium turbidity | Clear, breezy |
| 115 | 27/11/2020; 11.45 | KO/PW | 51.316764-1.203014 | 220ml | Low turbidity | Overcast, 6 degrees |
| 131 | 27/11/2020; 10.30 | KO/PW | 51.314365-1.206790 | 500ml | Low turbidity | Overcast, 6 degrees |
| 135 | 17/11/2020; 10.20 | KO/PW | 51.320395-1.207171 | 300ml | Low turbidity | Sunny, 12 degrees |
| 136 | 16/11/2020; 10.55 | KC/PW | 51.321417-1.207495 | 136/1: 320ml; 136/2: 180ml | Low turbidity | Overcast, dry, 12 degrees |
| 146 | 16/11/2020; 13.05 | KC/PW | 51.320626-1.20948 | 146/1:220ml; 146/2: 200ml | Low turbidity | Overcast, dry, 12 degrees |
| 153 | 27/11/2020; 14.30 | KO/PW | 51.320135-1.209509 | 350ml | Low turbidity | Overcast, 7 degrees |
| 155 | 27/11/2020; 14.00 | KO/PW | 51.321650-1.209372 | 250ml | Low turbidity | Overcast, 6 degrees |
| 161 | 17/11/2020; 11.30 | KO/PW | 51.320258-1.211856 | 500ml | Low turbidity | Sunny spells, 12 degrees |

Table S1 Stodmarsh NNR ditch samples
