# Supplementary material for: A qPCR assay for the rapid and specific detection of Shining ram’s-horn snail (Segmentina nitida) eDNA from Stodmarsh National Nature Reserve, UK
Source: PLoS One. 2023 Nov 15;18(11):e0288267. doi: 10.1371/journal.pone.0288267 (PMC10651049; doi:10.1371/journal.pone.0288267)
Supplement: S2 Table — (DOCX) [file pone.0288267.s004.docx]

| **Sample ID** | **Collection date/time** | **Sampler** | **What 3 words Location** | **Volume filtered** | **Sample Condition** | **Site conditions** |
| --- | --- | --- | --- | --- | --- | --- |
| 1 | 31/01/21 | BM | Unguarded-Grove-Decanter | 420ml | Low turbidity | Overcast, 1°C |
| 2 | 31/01/21 | BM | Chap-City-Decanter | 90ml | Low turbidity | Overcast, 1°C |
| 3 | 31/01/21 | BM | Cassettes-Legroom-Housing | 60ml | Low turbidity | Overcast, 1°C |
| 4 | 31/01/21 | BM | Rattled-Games-Foresight | 60ml | Low turbidity | Overcast, 1°C |
| 5 | 31/01/21 | BM | Observers-Rises-Stoppage | 200ml | Low turbidity | Overcast, 1°C |
| D1 | 10/01/21; 10.00 | HR | Happily-Zealous-Mixes | 240ml | Low turbidity | Overcast, 1°C |
| D2 | 10/01/21; 10.10 | HR | Processor-Symphonic-Winded | 240ml | Low turbidity | Overcast, 1°C |
| Top | 04/02/21; 13.05 | HR | Repayment-Trail-Occupiers | 85ml | Low turbidity | Sunny, light breeze 6°C |
| LHS | 04/02/21: 12.55 | HR | Mush-Arrive-Squad | 130ml | Medium turbidity | Sunny, light breeze, 6°C |
| RHS | 04/02/21: 12.50 | HR | Smokers-Sprouted- Copiers | 65ml | Medium turbidity | Sunny, light breeze, 6°C |

Table S2 Leicestershire ditch samples
