# Supplementary material for: A qPCR assay for the rapid and specific detection of Shining ram’s-horn snail (Segmentina nitida) eDNA from Stodmarsh National Nature Reserve, UK
Source: PLoS One. 2023 Nov 15;18(11):e0288267. doi: 10.1371/journal.pone.0288267 (PMC10651049; doi:10.1371/journal.pone.0288267)
Supplement: S3 Table — (DOCX) [file pone.0288267.s005.docx]

| **SPECIES** | **Ditch no.** | **Grid Ref** | **Date** | **Sampled by** | **Relative Abundance** |
| --- | --- | --- | --- | --- | --- |
| *Bathyomphalus contortus* | 34 | TR 22569 62165 | 18/12/20 | DJB | Common |
| *Bithynia leachii* | 34 | TR 22569 62165 | 18/12/20 | DJB | Frequent |
| *Bithynia tentaculata* | 34 | TR 22569 62165 | 18/12/20 | DJB | Frequent |
| *Lymnaea balthica (peregra)* | 34 | TR 22569 62165 | 18/12/20 | DJB | Common |
| *Lymnaea palustris/fusca* | 34 | TR 22569 62165 | 18/12/20 | DJB | Occasional |
| *Lymnaea stagnalis* | 34 | TR 22569 62165 | 18/12/20 | DJB | Occasional |
| *Physa fontinalis* | 34 | TR 22569 62165 | 18/12/20 | DJB | Common |
| *Planorbarius corneus* | 34 | TR 22569 62165 | 18/12/20 | DJB | Rare |
| *Planorbis planorbis* | 34 | TR 22569 62165 | 18/12/20 | DJB | Common |
| *Segmentina nitida* | 34 | TR 22569 62165 | 18/12/20 | DJB | Occasional |
| *Valvata cristata* | 34 | TR 22569 62165 | 18/12/20 | DJB | Occasional |
| *Anisus vortex* | 42 | TR 22650 61921 | 18/12/20 | DJB | Abundant |
| *Bathyomphalus contortus* | 42 | TR 22650 61921 | 18/12/20 | DJB | Frequent |
| *Bithynia leachii* | 42 | TR 22650 61921 | 18/12/20 | DJB | Occasional |
| *Bithynia tentaculata* | 42 | TR 22650 61921 | 18/12/20 | DJB | Occasional |
| *Lymnaea balthica (peregra)* | 42 | TR 22650 61921 | 18/12/20 | DJB | Abundant |
| *Lymnaea palustris/fusca* | 42 | TR 22650 61921 | 18/12/20 | DJB | Rare |
| *Lymnaea stagnalis* | 42 | TR 22650 61921 | 18/12/20 | DJB | Rare |
| *Physa fontinalis* | 42 | TR 22650 61921 | 18/12/20 | DJB | Abundant |
| *Planorbarius corneus* | 42 | TR 22650 61921 | 18/12/20 | DJB | Rare |
| *Planorbis carinatus* | 42 | TR 22650 61921 | 18/12/20 | DJB | Frequent |
| *Anisus vortex* | 44 | TR 22648 62002 | 18/12/20 | DJB | Common |
| *Bathyomphalus contortus* | 44 | TR 22648 62002 | 18/12/20 | DJB | Common |
| *Bithynia leachii* | 44 | TR 22648 62002 | 18/12/20 | DJB | Frequent |
| *Bithynia tentaculata* | 44 | TR 22648 62002 | 18/12/20 | DJB | Frequent |
| *Lymnaea balthica (peregra)* | 44 | TR 22648 62002 | 18/12/20 | DJB | Common |
| *Lymnaea palustris/fusca* | 44 | TR 22648 62002 | 18/12/20 | DJB | Frequent |
| *Physa fontinalis* | 44 | TR 22648 62002 | 18/12/20 | DJB | Frequent |
| *Planorbarius corneus* | 44 | TR 22648 62002 | 18/12/20 | DJB | Rare |
| *Planorbis carinatus* | 44 | TR 22648 62002 | 18/12/20 | DJB | Rare |
| *Planorbis planorbis* | 44 | TR 22648 62002 | 18/12/20 | DJB | Common |
| *Anisus vortex* | 56 | TR 22851 62073 | 18/12/20 | DJB | Frequent |
| *Bathyomphalus contortus* | 56 | TR 22851 62073 | 18/12/20 | DJB | Common |
| *Bithynia leachii* | 56 | TR 22851 62073 | 18/12/20 | DJB | Rare |
| *Bithynia tentaculata* | 56 | TR 22851 62073 | 18/12/20 | DJB | Frequent |
| *Hippeutis complanata* | 56 | TR 22851 62073 | 18/12/20 | DJB | Rare |
| *Lymnaea balthica (peregra)* | 56 | TR 22851 62073 | 18/12/20 | DJB | Frequent |
| *Lymnaea palustris/fusca* | 56 | TR 22851 62073 | 18/12/20 | DJB | Rare |
| *Physa fontinalis* | 56 | TR 22851 62073 | 18/12/20 | DJB | Common |
| *Planorbarius corneus* | 56 | TR 22851 62073 | 18/12/20 | DJB | Rare |
| *Planorbis planorbis* | 56 | TR 22851 62073 | 18/12/20 | DJB | Common |
| *Segmentina nitida* | 56 | TR 22851 62073 | 18/12/20 | DJB | Occasional |
| *Valvata cristata* | 56 | TR 22851 62073 | 18/12/20 | DJB | Occasional |
| *Anisus vortex* | 58 | TR 22863 61997 | 18/12/20 | DJB | Abundant |
| *Bathyomphalus contortus* | 58 | TR 22863 61997 | 18/12/20 | DJB | Common |
| *Bithynia leachii* | 58 | TR 22863 61997 | 18/12/20 | DJB | Occasional |
| *Bithynia tentaculata* | 58 | TR 22863 61997 | 18/12/20 | DJB | Frequent |
| *Gyraulus albus* | 58 | TR 22863 61997 | 18/12/20 | DJB | Rare |
| *Lymnaea balthica (peregra)* | 58 | TR 22863 61997 | 18/12/20 | DJB | Frequent |
| *Lymnaea palustris/fusca* | 58 | TR 22863 61997 | 18/12/20 | DJB | Rare |
| *Physa fontinalis* | 58 | TR 22863 61997 | 18/12/20 | DJB | Common |
| *Planorbis carinatus* | 58 | TR 22863 61997 | 18/12/20 | DJB | Frequent |
| *Planorbis planorbis* | 58 | TR 22863 61997 | 18/12/20 | DJB | Common |
| *Anisus vortex* | 60 | TR 22922 62351 | 17/12/20 | DJB | Common |
| *Bithynia leachii* | 60 | TR 22922 62351 | 17/12/20 | DJB | Common |
| *Bithynia tentaculata* | 60 | TR 22922 62351 | 17/12/20 | DJB | Common |
| *Lymnaea balthica (peregra)* | 60 | TR 22922 62351 | 17/12/20 | DJB | Abundant |
| *Lymnaea palustris/fusca* | 60 | TR 22922 62351 | 17/12/20 | DJB | Occasional |
| *Lymnaea stagnalis* | 60 | TR 22922 62351 | 17/12/20 | DJB | Rare |
| *Planorbarius corneus* | 60 | TR 22922 62351 | 17/12/20 | DJB | Rare |
| *Planorbis carinatus* | 60 | TR 22922 62351 | 17/12/20 | DJB | Rare |
| *Planorbis planorbis* | 60 | TR 22922 62351 | 17/12/20 | DJB | Occasional |
| *Anisus vortex* | 62 | TR 22922 62130 | 17/12/20 | DJB | Frequent |
| *Bathyomphalus contortus* | 62 | TR 22922 62130 | 17/12/20 | DJB | Frequent |
| *Bithynia leachii* | 62 | TR 22922 62130 | 17/12/20 | DJB | Frequent |
| *Bithynia tentaculata* | 62 | TR 22922 62130 | 17/12/20 | DJB | Frequent |
| *Hippeutis complanata* | 62 | TR 22922 62130 | 17/12/20 | DJB | Rare |
| *Lymnaea balthica (peregra)* | 62 | TR 22922 62130 | 17/12/20 | DJB | Frequent |
| *Lymnaea palustris/fusca* | 62 | TR 22922 62130 | 17/12/20 | DJB | Rare |
| *Lymnaea stagnalis* | 62 | TR 22922 62130 | 17/12/20 | DJB | Rare |
| *Physa fontinalis* | 62 | TR 22922 62130 | 17/12/20 | DJB | Frequent |
| *Planorbarius corneus* | 62 | TR 22922 62130 | 17/12/20 | DJB | Rare |
| *Planorbis carinatus* | 62 | TR 22922 62130 | 17/12/20 | DJB | Frequent |
| *Planorbis planorbis* | 62 | TR 22922 62130 | 17/12/20 | DJB | Frequent |
| *Segmentina nitida* | 62 | TR 22922 62130 | 17/12/20 | DJB | Occasional |
| *Valvata cristata* | 62 | TR 22922 62130 | 17/12/20 | DJB | Frequent |
| *Anisus vortex* | 65 | TR 22932 62063 | 17/12/20 | DJB | Occasional |
| *Bathyomphalus contortus* | 65 | TR 22932 62063 | 17/12/20 | DJB | Common |
| *Bithynia tentaculata* | 65 | TR 22932 62063 | 17/12/20 | DJB | Frequent |
| *Lymnaea balthica (peregra)* | 65 | TR 22932 62063 | 17/12/20 | DJB | Frequent |
| *Lymnaea palustris/fusca* | 65 | TR 22932 62063 | 17/12/20 | DJB | Rare |
| *Lymnaea stagnalis* | 65 | TR 22932 62063 | 17/12/20 | DJB | Rare |
| *Physa fontinalis* | 65 | TR 22932 62063 | 17/12/20 | DJB | Occasional |
| *Planorbarius corneus* | 65 | TR 22932 62063 | 17/12/20 | DJB | Rare |
| *Planorbis planorbis* | 65 | TR 22932 62063 | 17/12/20 | DJB | Common |
| *Segmentina nitida* | 65 | TR 22932 62063 | 17/12/20 | DJB | Occasional |
| *Valvata cristata* | 65 | TR 22932 62063 | 17/12/20 | DJB | Rare |
| *Bathyomphalus contortus* | 70 | TR 22978 62130 | 17/12/20 | DJB | Common |
| *Bithynia leachii* | 70 | TR 22978 62130 | 17/12/20 | DJB | Occasional |
| *Bithynia tentaculata* | 70 | TR 22978 62130 | 17/12/20 | DJB | Frequent |
| *Hippeutis complanata* | 70 | TR 22978 62130 | 17/12/20 | DJB | Rare |
| *Lymnaea balthica (peregra)* | 70 | TR 22978 62130 | 17/12/20 | DJB | Common |
| *Lymnaea palustris/fusca* | 70 | TR 22978 62130 | 17/12/20 | DJB | Common |
| *Lymnaea stagnalis* | 70 | TR 22978 62130 | 17/12/20 | DJB | Rare |
| *Physa fontinalis* | 70 | TR 22978 62130 | 17/12/20 | DJB | Common |
| *Planorbarius corneus* | 70 | TR 22978 62130 | 17/12/20 | DJB | Occasional |
| *Planorbis planorbis* | 70 | TR 22978 62130 | 17/12/20 | DJB | Occasional |
| *Segmentina nitida* | 70 | TR 22978 62130 | 17/12/20 | DJB | Rare |
| *Valvata cristata* | 70 | TR 22978 62130 | 17/12/20 | DJB | Occasional |
| *Bathyomphalus contortus* | 87 | TR 23140 62365 | 27/01/21 | DJB | Frequent |
| *Bithynia leachii* | 87 | TR 23140 62365 | 27/01/21 | DJB | Occasional |
| *Bithynia tentaculata* | 87 | TR 23140 62365 | 27/01/21 | DJB | Occasional |
| *Hippeutis complanata* | 87 | TR 23140 62365 | 27/01/21 | DJB | Rare |
| *Lymnaea balthica (peregra)* | 87 | TR 23140 62365 | 27/01/21 | DJB | Frequent |
| *Lymnaea palustris/fusca* | 87 | TR 23140 62365 | 27/01/21 | DJB | Rare |
| *Physa fontinalis* | 87 | TR 23140 62365 | 27/01/21 | DJB | Rare |
| *Planorbarius corneus* | 87 | TR 23140 62365 | 27/01/21 | DJB | Rare |
| *Planorbis planorbis* | 87 | TR 23140 62365 | 27/01/21 | DJB | Frequent |
| *Segmentina nitida* | 87 | TR 23140 62365 | 27/01/21 | DJB | Frequent |
| *Bathyomphalus contortus* | 92 | TR 23180 62323 | 27/01/21 | DJB | Frequent |
| *Bithynia leachii* | 92 | TR 23180 62323 | 27/01/21 | DJB | Rare |
| *Bithynia tentaculata* | 92 | TR 23180 62323 | 27/01/21 | DJB | Occasional |
| *Lymnaea palustris/fusca* | 92 | TR 23180 62323 | 27/01/21 | DJB | Occasional |
| *Physa fontinalis* | 92 | TR 23180 62323 | 27/01/21 | DJB | Frequent |
| *Planorbis planorbis* | 92 | TR 23180 62323 | 27/01/21 | DJB | Occasional |
| *Segmentina nitida* | 92 | TR 23180 62323 | 27/01/21 | DJB | Frequent |
| *Anisus vortex* | 98 | TR 23224 62163 | 22/12/20 | DJB | Occasional |
| *Bathyomphalus contortus* | 98 | TR 23224 62163 | 22/12/20 | DJB | Common |
| *Bithynia leachii* | 98 | TR 23224 62163 | 22/12/20 | DJB | Frequent |
| *Bithynia tentaculata* | 98 | TR 23224 62163 | 22/12/20 | DJB | Occasional |
| *Lymnaea balthica (peregra)* | 98 | TR 23224 62163 | 22/12/20 | DJB | Occasional |
| *Lymnaea palustris/fusca* | 98 | TR 23224 62163 | 22/12/20 | DJB | Occasional |
| *Physa fontinalis* | 98 | TR 23224 62163 | 22/12/20 | DJB | Rare |
| *Planorbis carinatus* | 98 | TR 23224 62163 | 22/12/20 | DJB | Occasional |
| *Planorbis planorbis* | 98 | TR 23224 62163 | 22/12/20 | DJB | Occasional |
| *Valvata cristata* | 98 | TR 23224 62163 | 22/12/20 | DJB | Occasional |
| *Anisus vortex* | 106 | TR 23325 62545 | 27/01/21 | DJB | Rare |
| *Bithynia leachii* | 106 | TR 23325 62545 | 27/01/21 | DJB | Rare |
| *Bithynia tentaculata* | 106 | TR 23325 62545 | 27/01/21 | DJB | Frequent |
| *Lymnaea balthica (peregra)* | 106 | TR 23325 62545 | 27/01/21 | DJB | Frequent |
| *Physa fontinalis* | 106 | TR 23325 62545 | 27/01/21 | DJB | Abundant |
| *Planorbis carinatus* | 106 | TR 23325 62545 | 27/01/21 | DJB | Occasional |
| *Planorbis planorbis* | 106 | TR 23325 62545 | 27/01/21 | DJB | Rare |
| *Anisus vortex* | 108 | TR 23306 62242 | 22/12/20 | DJB | Frequent |
| *Bathyomphalus contortus* | 108 | TR 23306 62242 | 22/12/20 | DJB | Frequent |
| *Bithynia leachii* | 108 | TR 23306 62242 | 22/12/20 | DJB | Frequent |
| *Bithynia tentaculata* | 108 | TR 23306 62242 | 22/12/20 | DJB | Frequent |
| *Lymnaea stagnalis* | 108 | TR 23306 62242 | 22/12/20 | DJB | Rare |
| *Physa fontinalis* | 108 | TR 23306 62242 | 22/12/20 | DJB | Frequent |
| *Planorbarius corneus* | 108 | TR 23306 62242 | 22/12/20 | DJB | Occasional |
| *Planorbis carinatus* | 108 | TR 23306 62242 | 22/12/20 | DJB | Frequent |
| *Planorbis planorbis* | 108 | TR 23306 62242 | 22/12/20 | DJB | Occasional |
| *Segmentina nitida* | 108 | TR 23306 62242 | 22/12/20 | DJB | Occasional |
| *Valvata cristata* | 108 | TR 23306 62242 | 22/12/20 | DJB | Occasional |
| *Anisus vortex* | 115 | TR 23369 62472 | 27/01/21 | DJB | Frequent |
| *Bathyomphalus contortus* | 115 | TR 23369 62472 | 27/01/21 | DJB | Rare |
| *Bithynia leachii* | 115 | TR 23369 62472 | 27/01/21 | DJB | Occasional |
| *Bithynia tentaculata* | 115 | TR 23369 62472 | 27/01/21 | DJB | Frequent |
| *Lymnaea balthica (peregra)* | 115 | TR 23369 62472 | 27/01/21 | DJB | Common |
| *Lymnaea palustris/fusca* | 115 | TR 23369 62472 | 27/01/21 | DJB | Rare |
| *Lymnaea stagnalis* | 115 | TR 23369 62472 | 27/01/21 | DJB | Rare |
| *Physa fontinalis* | 115 | TR 23369 62472 | 27/01/21 | DJB | Common |
| *Planorbarius corneus* | 115 | TR 23369 62472 | 27/01/21 | DJB | Rare |
| *Planorbis carinatus* | 115 | TR 23369 62472 | 27/01/21 | DJB | Frequent |
| *Planorbis planorbis* | 115 | TR 23369 62472 | 27/01/21 | DJB | Rare |
| *Valvata cristata* | 115 | TR 23369 62472 | 27/01/21 | DJB | Frequent |
| *Anisus vortex* | 131 | TR 23516 62109 | 22/12/20 | DJB | Occasional |
| *Bathyomphalus contortus* | 131 | TR 23516 62109 | 22/12/20 | DJB | Common |
| *Bithynia leachii* | 131 | TR 23516 62109 | 22/12/20 | DJB | Occasional |
| *Bithynia tentaculata* | 131 | TR 23516 62109 | 22/12/20 | DJB | Frequent |
| *Lymnaea palustris/fusca* | 131 | TR 23516 62109 | 22/12/20 | DJB | Rare |
| *Lymnaea stagnalis* | 131 | TR 23516 62109 | 22/12/20 | DJB | Occasional |
| *Physa fontinalis* | 131 | TR 23516 62109 | 22/12/20 | DJB | Occasional |
| *Planorbarius corneus* | 131 | TR 23516 62109 | 22/12/20 | DJB | Occasional |
| *Planorbis carinatus* | 131 | TR 23516 62109 | 22/12/20 | DJB | Frequent |
| *Planorbis planorbis* | 131 | TR 23516 62109 | 22/12/20 | DJB | Common |
| *Anisus vortex* | 135 | TR 23568 62793 | 22/12/20 | DJB | Frequent |
| *Bathyomphalus contortus* | 135 | TR 23568 62793 | 22/12/20 | DJB | Occasional |
| *Bithynia tentaculata* | 135 | TR 23568 62793 | 22/12/20 | DJB | Occasional |
| *Hippeutis complanata* | 135 | TR 23568 62793 | 22/12/20 | DJB | Rare |
| *Lymnaea balthica (peregra)* | 135 | TR 23568 62793 | 22/12/20 | DJB | Frequent |
| *Lymnaea palustris/fusca* | 135 | TR 23568 62793 | 22/12/20 | DJB | Rare |
| *Lymnaea stagnalis* | 135 | TR 23568 62793 | 22/12/20 | DJB | Occasional |
| *Physa fontinalis* | 135 | TR 23568 62793 | 22/12/20 | DJB | Frequent |
| *Planorbis carinatus* | 135 | TR 23568 62793 | 22/12/20 | DJB | Frequent |
| *Planorbis planorbis* | 135 | TR 23568 62793 | 22/12/20 | DJB | Frequent |
| *Valvata cristata* | 135 | TR 23568 62793 | 22/12/20 | DJB | Occasional |
| *Bathyomphalus contortus* | 136 | TR 23583 62913 | 15/12/20 | DJB | Rare |
| *Bithynia leachii* | 136 | TR 23583 62913 | 15/12/20 | DJB | Common |
| *Bithynia tentaculata* | 136 | TR 23583 62913 | 15/12/20 | DJB | Common |
| *Gyraulus crista* | 136 | TR 23583 62913 | 15/12/20 | DJB | Rare |
| *Lymnaea balthica (peregra)* | 136 | TR 23583 62913 | 15/12/20 | DJB | Common |
| *Physa fontinalis* | 136 | TR 23583 62913 | 15/12/20 | DJB | Frequent |
| *Planorbis planorbis* | 136 | TR 23583 62913 | 15/12/20 | DJB | Frequent |
| *Bithynia tentaculata* | 146 | TR 23674 62820 | 27/01/21 | DJB | Occasional |
| *Lymnaea balthica (peregra)* | 146 | TR 23674 62820 | 27/01/21 | DJB | Frequent |
| *Physa fontinalis* | 146 | TR 23674 62820 | 27/01/21 | DJB | Common |
| *Planorbis planorbis* | 146 | TR 23674 62820 | 27/01/21 | DJB | Rare |
| *Bithynia tentaculata* | 153 | TR 23802 62806 | 27/01/21 | DJB | Frequent |
| *Lymnaea balthica (peregra)* | 153 | TR 23802 62806 | 27/01/21 | DJB | Frequent |
| *Lymnaea palustris/fusca* | 153 | TR 23802 62806 | 27/01/21 | DJB | Rare |
| *Physa fontinalis* | 153 | TR 23802 62806 | 27/01/21 | DJB | Common |
| *Planorbis carinatus* | 153 | TR 23802 62806 | 27/01/21 | DJB | Occasional |
| *Planorbis planorbis* | 153 | TR 23802 62806 | 27/01/21 | DJB | Occasional |
| *Bithynia tentaculata* | 155 | TR 23810 62930 | 27/01/21 | DJB | Rare |
| *Gyraulus crista* | 155 | TR 23810 62930 | 27/01/21 | DJB | Abundant |
| *Lymnaea balthica (peregra)* | 155 | TR 23810 62930 | 27/01/21 | DJB | Common |
| *Anisus vortex* | 161 | TR 23889 62771 | 16/12/20 | DJB | Frequent |
| *Bithynia tentaculata* | 161 | TR 23889 62771 | 16/12/20 | DJB | Frequent |
| *Planorbis carinatus* | 161 | TR 23889 62771 | 16/12/20 | DJB | Frequent |
| *Planorbis planorbis* | 161 | TR 23889 62771 | 16/12/20 | DJB | Frequent |

Table S4 Mollusc species list as sampled by Dan Bennett (Dec 2020). The abundance attribution made by D. Bennett is qualitative.
