# Supplementary material for: A qPCR assay for the rapid and specific detection of Shining ram’s-horn snail (Segmentina nitida) eDNA from Stodmarsh National Nature Reserve, UK
Source: PLoS One. 2023 Nov 15;18(11):e0288267. doi: 10.1371/journal.pone.0288267 (PMC10651049; doi:10.1371/journal.pone.0288267)
Supplement: S4 Table — Where there was poor PCR amplification/sequence we were unable to amplify sufficient target gene to enable us to identify the species and the specimen was not used further. Valvata cristata samples only showed 96 or 97% identity to those on the NCBI database with the sequences also being found to be similarly close to those of Valvata relicta which is not found in the UK and is therefore unlikely to be present. The sequences were manually checked versus their respective chromatograms to resolve any errors in base calling prior to identification via BLAST searches, however, no improvements were made. (DOCX) [file pone.0288267.s006.docx]

| Snail ID | Sample ID | DNA (ng/ul) | Eluted volume (ul) | Confirmed sequence ID |
| --- | --- | --- | --- | --- |
| *Gyraulus crista* | 1 | 4.68 | 200 | Yes |
| *Gyraulus crista* | 1a | 29.4 | 50 | Yes |
| *Planorbis planorbis* | 2 | 2.81 | 200 | Yes |
| *Planorbis planorbis* | 2a | 39.9 | 50 | Yes |
| *Bathyomphalus contortus* | 3a | 10.6 | 50 | Yes |
| *Hippeutis complanatus* | 4 | 3.58 | 200 | Yes |
| *Hippeutis complanatus* | 4a | 11.8 | 50 | Yes |
| *Planorbis carinatus* | 5a | 22.4 | 50 | Yes |
| *Planorbis carinatus* | 5b | 38.6 | 50 | Yes |
| *Planorbarius corneus* | 6 | 9.35 | 200 | Yes |
| *Planorbarius corneus* | 6a | 29.7 | 50 | Yes |
| *Segmentina nitida* | 7 | 8.52 | 200 | Yes |
| *Segmentina nitida* | 7a | 29.6 | 50 | Yes |
| *Segmentina nitida* | 7b | 46.4 | 50 | Yes |
| *Segmentina nitida* | 7c | 12.5 | 50 | Yes |
| *Anisus vortex* | 8a | 33.1 | 50 | Yes |
| *Valvata cristata* | 9 | 1.59 | 200 | Yes (97%) |
| *Valvata cristata* | 9a | 28 | 50 | Yes (96%) |
| *Valvata cristata* | 9b | 30.9 | 50 | Yes (97%) |
| *Gyraulus albus* | 10 | 2.42 | 200 | Yes |

Table S5. DNA information per snail species. Where there was poor PCR amplification/sequence we were unable to amplify sufficient target gene to enable us to identify the species and the specimen was not used further.

*Valvata cristata* samples only showed 96 or 97% identity to those on the NCBI database with the sequences also being found to be similarly close to those of *Valvata relicta* which is not found in the UK and is therefore unlikely to be present. The sequences were manually checked versus their respective chromatograms to resolve any errors in base calling prior to identification via BLAST searches, however, no improvements were made.
