# Supplementary material for: A qPCR assay for the rapid and specific detection of Shining ram’s-horn snail (Segmentina nitida) eDNA from Stodmarsh National Nature Reserve, UK
Source: PLoS One. 2023 Nov 15;18(11):e0288267. doi: 10.1371/journal.pone.0288267 (PMC10651049; doi:10.1371/journal.pone.0288267)
Supplement: S5 Table — (DOCX) [file pone.0288267.s007.docx]

| Primer Pair | Forward Primer Sequence (5’ to 3’) | Reverse Primer Sequence (5’ to 3’) | Probe Sequence (5’ FAM to 3’ BHQ-1) | Product Length | Cross-species amplification |
| --- | --- | --- | --- | --- | --- |
| 1 | GGAGGTGTTGGTACTGGGTG | GTAATACCTGGAGCCCGCAT | GCGGTCCTATTGCACATGGTGGT | 173 | Yes |
| 2 | AGGAGGTGTTGGTACTGGGT | CCTGGAGCCCGCATGTTTAT | GCGGTCCTATTGCACATGGTGGT | 168 | No |
| 3 | CATGCGGGCTCCAGGTATTA | CCACCACCTGCTGGATCAAA | ACCAGTTTTAGCTGGTGCCATTACAA | 168 | Yes |
| 4 | ATAAACATGCGGGCTCCAGG | TAGGATCACCACCACCTGCT | ACCAGTTTTAGCTGGTGCCATTACAA | 181 | No |
| 5 | CCTATTGCACATGGTGGTGC | ACCACCTGCTGGATCAAAGA | ACATGCGGGCTCCAGGTATTACT | 273 | Yes |
| 6 | TGCGGGCTCCAGGTATTACT | ATAGGATCACCACCACCTGC | ACCAGTTTTAGCTGGTGCCATTACAA | 175 | Yes |
| 7 | GAAGGAGGTGTTGGTACTGGG | AGTAATACCTGGAGCCCGCA | GCGGTCCTATTGCACATGGTGGT | 177 | Yes |
| 8 | GCGGTCCTATTGCACATGGT | CACCACCTGCTGGATCAAAG | ACATGCGGGCTCCAGGTATTACT | 279 | Yes |
| 9 | CCACTTTTAATTGGGGCTCCG | CCATGTGCAATAGGACCGCT | TGAAGGAGGTGTTGGTACTGGGTG | 167 | No |
| 10 | GAGGTGTTGGTACTGGGTGA | CCTGGAGCCCGCATGTTTA | GCGGTCCTATTGCACATGGTGGT | 166 | No |

Table S7 Ten potential primer/probe combinations for S. nitida species-specific PCR for in silico testing.
