# Supplementary material for: A qPCR assay for the rapid and specific detection of Shining ram’s-horn snail (Segmentina nitida) eDNA from Stodmarsh National Nature Reserve, UK
Source: PLoS One. 2023 Nov 15;18(11):e0288267. doi: 10.1371/journal.pone.0288267 (PMC10651049; doi:10.1371/journal.pone.0288267)
Supplement: S1 Protocol — (DOCX) [file pone.0288267.s008.docx]

# S3 Protocol Collection of Invertebrate Samples

1. Using a Freshwater Biological Association design, rectangular frame pond net (20-25 cm x 19-22 cm x 30 cm deep) with a 1 mm mesh, each ditch was pond netted three times from the bankside. Vegetation was netted by making short jabbing thrusts into dense emergent and raft-forming plants, making occasional longer strokes into submerged plants and over bare substrate in deeper water. Patches of vegetation that exhibit the greatest small-scale mosaic structure were selected since these patches yield more specimens. Netting stopped after 1 to 3 minutes when the net began to fill to the point where it becomes difficult to push and is usually a quarter to a third full of plant material (about 2 to 3 litres by volume). Careful manipulation slowed the rate at which algae and duckweed were caught while probing more productive structures. Bottom sediment was avoided since it clogs the net and contains almost no species that contribute to the analysis.
2. Bank sorting was carried out for each haul for 10 minutes, giving 30 minutes of sorting for each ditch. The sample was tipped onto a white plastic sheet and spread into a thin layer. Fast crawling beetles, bugs and dragonfly larvae are collected or identified (if recognisable) before they escape during the spreading-out process. The sheet is then scanned for other animals. Species identified in the field were not collected but recorded in situ and released back to the ditch. Fine flexible forceps were used for picking up animals, collecting one or two individuals of each different taxa encountered and placing them in ethanol (70%) for identification in the lab if required.
3. After sorting and picking over on the plastic sheet, the material from the sheet was tipped into a large bucket with some ditch water, combining the catch from the three hauls taken from a particular ditch. To find any weakly swimming animals and molluscs two final operations were carried out. Part of the debris was put into a large, strong, white tray with 1-2cm of water so that feeble animals can swim free, and were collected, and the tray emptied back into the bucket.
4. Finally, the plant material was swished about in the bucket of water, the larger pieces removed, and most of the water decanted, and then the heavy residue was tipped into a large, strong, white tray with around 1 cm of water. By tilting the tray slowly back and forth, carefully pouring off excess water from one corner, the molluscs were left stranded in a pile because they sink, enabling identification in the field and/or collection for preservation where required.
5. Specimens of *Segmentina nitida* and any similar species (ram’s-horn shaped) if encountered such as *Hippeutis complanatus, Anisus vortex, Anisus spirorbis, Planorbis planorbis, Planorbis carinatus, Bathyomphalus contortus and Gyraulus sp*. were collected for the DNA analysis and development of primers for the single species assay. These were separated into sterile tubes, containing 95% ethanol, with one species only per tube, approximately five individuals for each species (not separated by ditch at this point) and couriered to the laboratory.
